# Thiourea‐Derived Single‐Source Molecular Precursor For Spin‐Coated PbS Thin Films

**DOI:** 10.1002/open.202300045

**Published:** 2023-04-14

**Authors:** Kevin I. Y. Ketchemen, Vaidehi Lapalikar, Eduardo Carrillo‐Aravena, Linda D. Nyamen, Peter T. Ndifon, Michael Ruck

**Affiliations:** ^1^ Faculty of Chemistry and Food Chemistry Technische Universität Dresden 01062 Dresden Germany; ^2^ Department of Inorganic Chemistry University of Yaoundé I P.O. Box 812 Yaoundé Cameroon; ^3^ Max Planck Institute for Chemical Physics of Solids (MPI-CPfS) Nöthnitzer Str. 40 01187 Dresden Germany

**Keywords:** crystal structure, molecular precursor, semiconductors, thin films, spin coating

## Abstract

In search of a suitable single‐source precursor for the deposition of nanostructured PbS thin films at moderate temperatures under ambient conditions, we have synthesized the ligand *N*‐(thiomorpholine‐4‐carbothioyl)benzamide and its corresponding lead(II) complex. The structures of both compounds were determined by single‐crystal X‐ray diffraction. In the complex, two ligands coordinate to a lead(II) atom in hemi‐directed geometry through S and O atoms. Secondary intermolecular Pb⋅⋅⋅S interactions group the complexes into pairs. As bulk powders, both the ligand and complex show nominal composition and purity as evidenced by elemental analysis, ^1^H NMR and IR spectroscopy. Thermal analysis of the lead(II) complex was carried out to understand its thermal decomposition behaviour for establishing a thin film fabrication protocol. Thin films of phase‐pure PbS were fabricated using this new molecular precursor at the comparatively low annealing temperature of 250 °C. The film showed nanoparticles with cuboidal morphology and a blue‐shifted optical absorption.

## Graphical abstract

**Lead coating**: The crystal structures of both *N*‐(thiomorpholine‐4‐carbothioyl)benzamide ligand and bis(*N*‐(thiomorpholine‐4‐carbothioyl)benzamidate)lead(II) complex are reported, and the usability of the complex as a single‐source molecular precursor for the deposition of PbS thin films via spin coating is demonstrated. The films show a blue shift in their absorption behavior and consist of particles with cuboidal morphology.

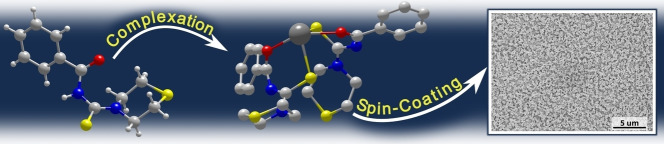

## Introduction

Metal chalcogenides have garnered tremendous attention in applications such as in sensors, photocatalysis, solar cells, etc. due to their robust chemical and thermal stability, tunable properties and diverse fabrication methods. Lead sulfide (PbS) is one such material from the class of IV–VI semiconductors.[^1^, ^2^, ^3^, ^4^, ^5^, ^6^] PbS possesses interesting physical and chemical properties in the nanoscale domain and displays unique quantization effects which are accompanied by a direct band gap of 0.41 eV at 300 K and a large excitonic Bohr radius of 18 nm.^[7]^ Several synthetic approaches have been used to synthesize this material in bulk as well as in thin films, resulting in a wide range of particle sizes and morphologies and a tunable bandgap from near‐infrared to the ultra‐violet.^[8]^ This tunability results in useful properties,[^9^, ^10^] making PbS suitable for application in solar cells,^[11]^ photocatalysis,[^12^, ^13^] gas sensors,^[14]^ tunable near‐infrared detectors,^[15]^ and light emitting diodes.^[16]^

Several methods of synthesis such as atmospheric‐ or low‐pressure metal‐organic chemical vapor deposition (AP or LP‐MOCVD),^[17]^ chemical bath deposition (CBD),^[18]^ reactive sputtering,^[19]^ spray pyrolysis,^[20]^ aerosol‐assisted chemical vapor deposition (AACVD)^[21]^ and spin coating,^[22]^ are used for the deposition of metal chalcogenide thin films. In this work, the spin coating method was used because it is a simple and cost‐effective technique that has a lot of potential for technological advancement. Thin film fabrication with a single molecular precursor has the advantage of providing a mixture of the film's constituents at the atomic level, resulting in films of high quality and purity, and is easier to handle than deposition with multiple precursors.[^1^, ^23^, ^24^, ^25^, ^26^, ^27^] Various families of inorganic compounds containing metal chalcogenides have been investigated for their suitability as precursors for the fabrication of high‐quality metal chalcogenides nanostructured materials.[^28^, ^29^, ^30^] Some examples include aminethiol,^[31]^ dithiocarbamate,^[32]^ thiobiuret,^[33]^ xanthate^[34]^ and thiourea.^[35]^

However, the key to the success of this technique lies in the nifty design of a molecular precursor that is, on one hand, sufficiently stable and storable and, on the other hand, decomposes into a pure solid at moderately elevated temperature without leaving any residues.[^3^, ^36^]

Heterocyclic thiourea compounds are potential good candidates for the fabrication of high‐quality metal sulfide nanomaterials. With these considerations in mind, we report herein the development of a new ligand and a new molecular precursor that can be employed to yield bulk powder or thin films of PbS under ambient conditions and at moderate temperatures. Specifically, we discuss in detail the synthesis and structure determination of the ligand *N*‐(thiomorpholine‐4‐carbothioyl)benzamide (**L1**) and its corresponding lead complex, bis(*N*‐(thiomorpholine‐4‐carbothioyl)benzamidate)lead(II) (**C1**), followed by microanalysis of the same. Furthermore, we show that **C1** can be used to fabricate thin films of PbS using the spin coating method at low annealing temperatures. Finally, we also report the morphological and optical properties of the deposited PbS thin films.

## Results and Discussion

### Structure determination of the ligand and the lead(II) complex

**Figure 1:**
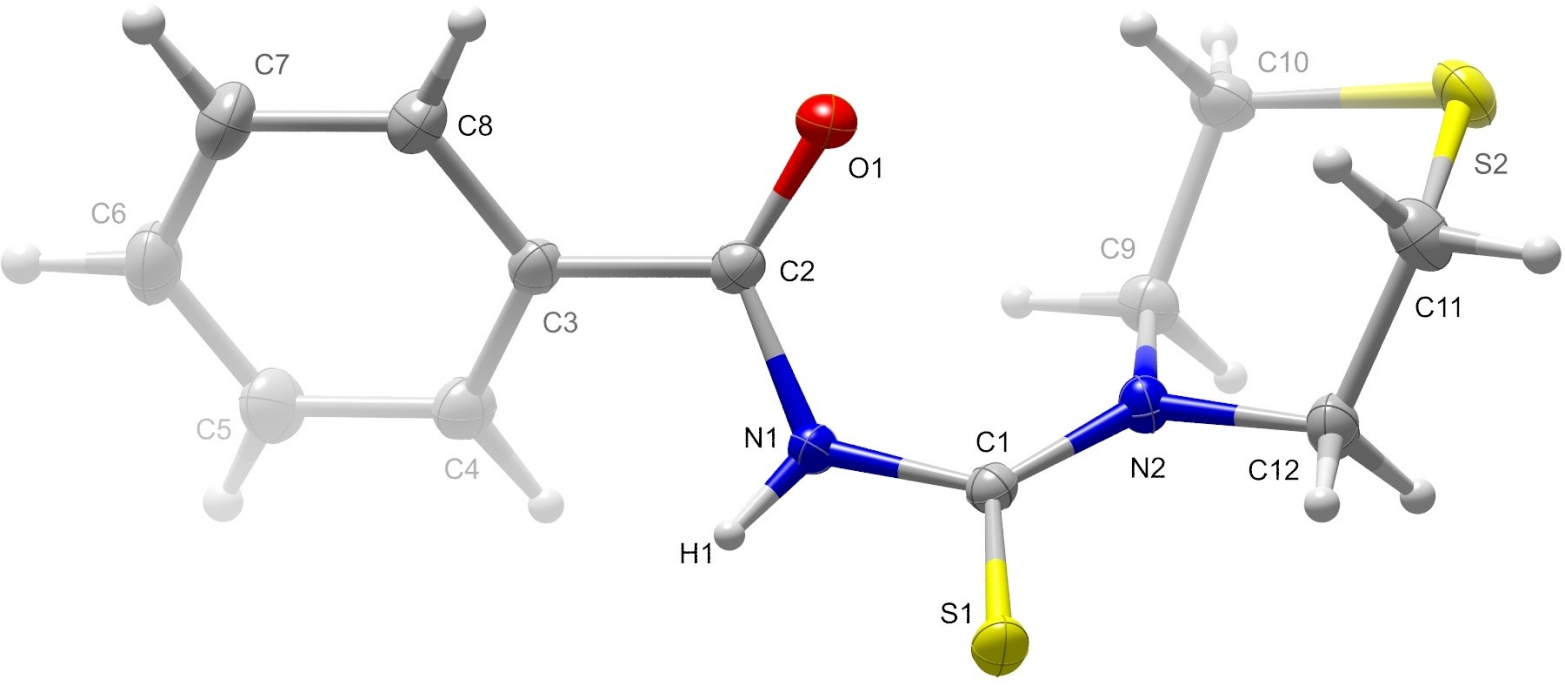
Molecule **L1** with atom labeling. Displacement ellipsoids of non‐hydrogen atoms comprise 70 % of the probability.

**Figure 2:**
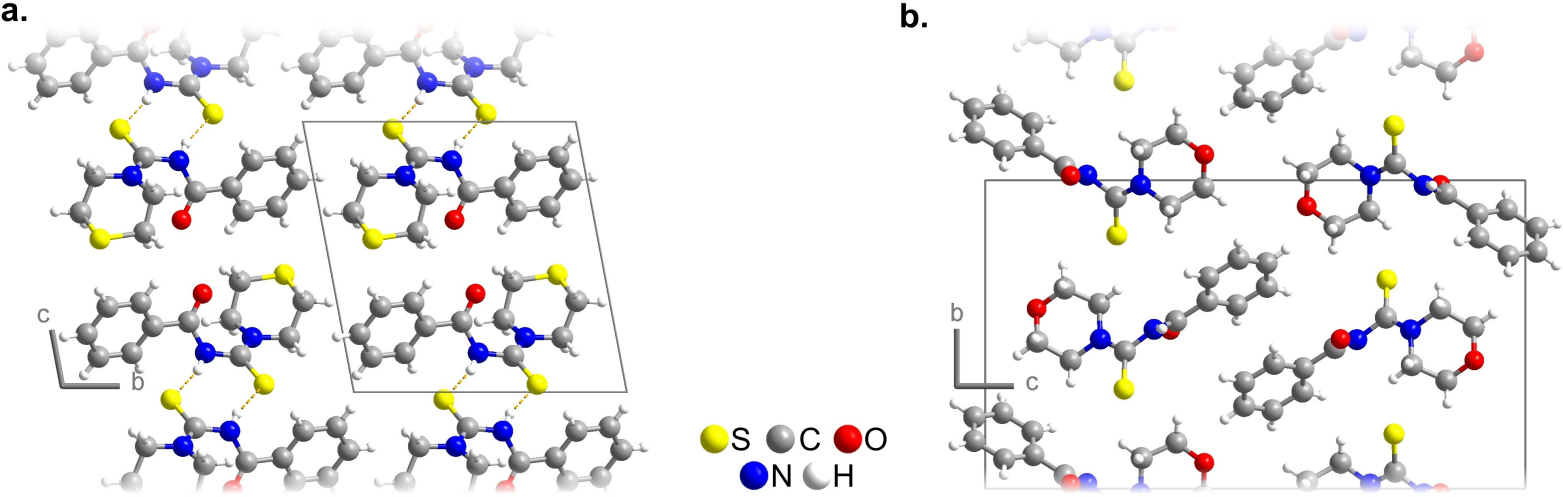
Crystal structures of **L1** (left) and **L2** (right) projected along their shortest crystallographic axes showing different packing sequences. In **L1**, hydrogen bonds connect molecules to form pairs, while in **L2**, they create chains along the viewing direction. The plot of **L2** was created from a new refinement based on deposited data.^[27]^

L1 can be understood as an acyl‐thiourea derivative (Figure 1). It crystallizes in the triclinic space group *P* $\overset{-}{1}$ and contains two molecules in the unit cell. Detailed crystallographic information can be found in Tables S1 to S5 (Supporting Information). As expected for a *N*‐(acyl)‐*N′*,*N′*‐(disubstituted)thiourea derivative, the S1=C⋅⋅⋅C=O torsion angle is anticlinal (115.51(6)°).[^37^, ^38^] The acidic hydrogen atom (H1) bonded to N1 bends out of the C1−N1−C2 plane by 58.5(6)° (distance H1‐plane: 4.5(1) pm) to create a hydrogen bond with the sulfur atom of the thiourea group of a neighboring molecule related by an inversion center (H1⋅⋅⋅S1 distance: 264(2) pm, N1−H1⋅⋅⋅S1 angle: 154(1)°), creating an eight‐membered ring including two hydrogen bridges (graph set $R_{2}^{2}(8$ ),^[39]^ Figure 2a).

We found a report of the crystal structure of the analogous ligand *N*‐(morpholine‐4‐carbothioyl)benzamide (**L2**) measured at room temperature.^[40]^ There, abnormally elongated thermal ellipsoids, especially for the aromatic carbon atoms, were resolved in a disorder model. Unfortunately, the acidic hydrogen atom of **L2** was refined using riding model constrained to trigonal planar coordination geometry at the nitrogen atom, making a comparison with the structure of **L1** impossible. A newer dataset of **L2** in the CCDC^[41]^ (Deposition Number: CCDC‐2100916) includes the refinement data at room temperature, which we used to perform a refinement to obtain comparable data (for SHELX input, see the Supporting Information). The carbon atoms in the aromatic ring were refined without splitting and showed good agreement with the ones obtained for **L1**. For the hydrogen atom bonded to the thiourea group, the riding model constraining was replaced by a softer DFIX restrain. This showed that the hydrogen atom bends away from the C1−N1−C2 plane by 66(3)° (distance from the plane: 36(4) pm), again, favoring the formation of a hydrogen bond with the carbonyl group from a neighboring molecule (H⋅⋅⋅O distance=212(3) pm, N−H⋅⋅⋅O angle: 167(3)°)), linking them into a zig‐zag chain that propagates along the [100] direction (graph set *C(4)*, Figure 2b).

**L1** and **L2** are closely related molecules that have similar torsion angles for the bond between the benzyl groups and the thiourea backbone (Table 1). However, there is a significant difference in the conformation of the (thio)morpholine ring. The packing of the molecules and the hydrogen bonding system also differ significantly (Figure 2).

**Table 1:** Selected torsion angles for **L1** and **L2**.

Torsion angle	**L1**	**L2**
S1−C1−C2−O1	115.5(6)°	103.2(3)°
N1−C2−C3−C4	−33.6(1)°	−33.5(5)°
C1−N2−C12−C11	−123.4(8)°	127.1(4)°

**Figure 3:**
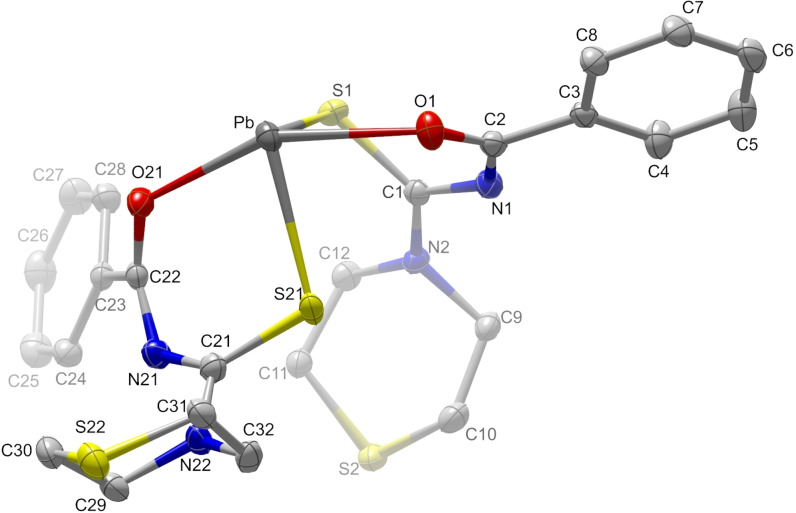
Asymmetric unit of the lead complex **C1** with numbering scheme. Displacement ellipsoids comprise 70 % of the probability. Hydrogen atoms were omitted for clarity.

**Figure 4:**
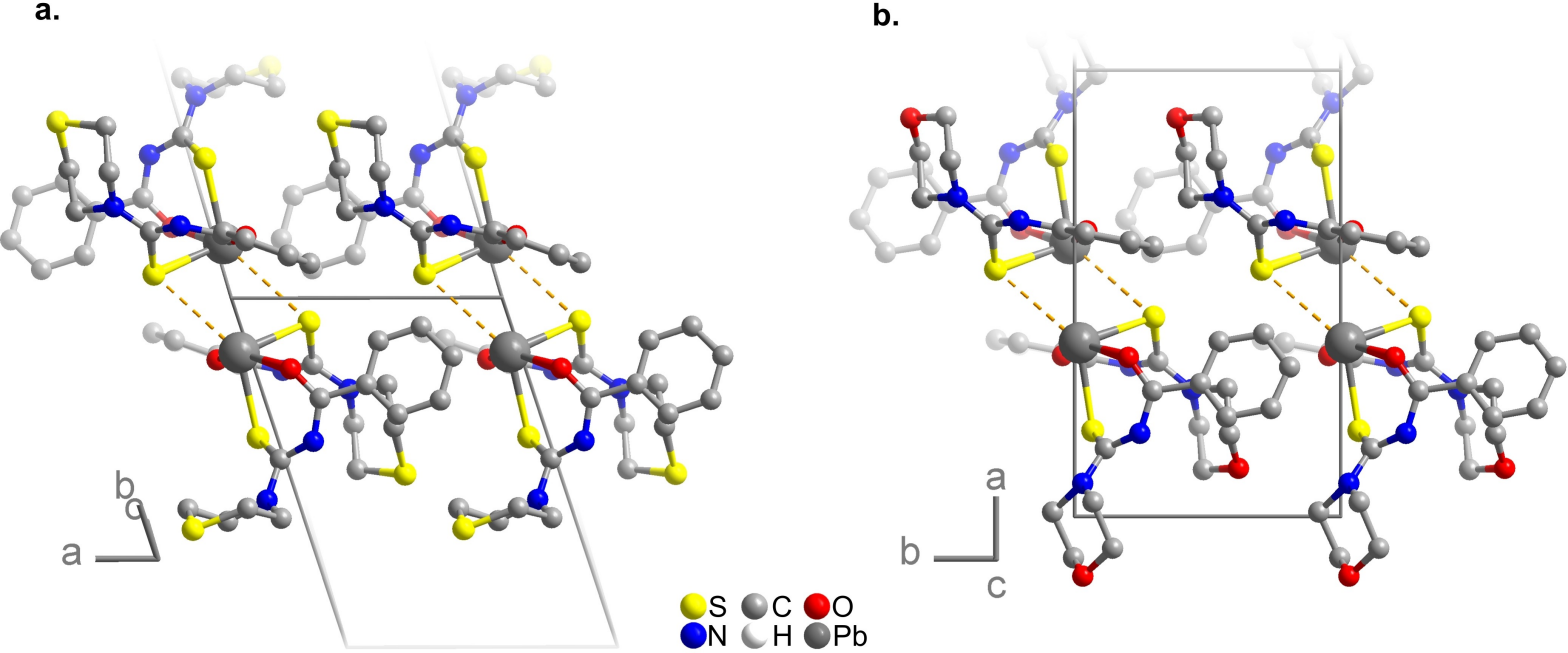
Packing of the lead complexes **C1** (a) and **C2** (b), showing the pairing of molecules. The plot of **C2** was created from deposited data.^[1]^ Hydrogen atoms were omitted for clarity.

The lead complex **C1** crystallizes in the triclinic space group *P* $\overset{-}{1}$ with two molecules in the unit cell related by a center of inversion. (Tables S1 and S6–S9 of the Supporting Information) Two *N*‐(thiomorpholine‐4‐carbothioyl)benzamidate anions coordinate the lead(II) cation via the oxygen and sulfur atoms of their carbonyl and carbothioyl groups, respectively (Figure 3). The coordination of the lead(II) cation shares great similarities with the one reported for the analogous, but not isostructural, morpholine complex bis(*N*‐(morpholine‐4‐carbothioyl)benzamidate)lead(II) (**C2**).^[1]^ The hemi‐directed sawhorse coordination geometry with S−Pb−S angles of 94.2(1)° and O−Pb−O angles of 150.0(1)° is typical for heavy p‐block elements of the periodic table. It can be rationalized by bonding through the 6p orbitals of lead using one or both orbital lobes, while the lone pair is predominantly 6 s in character, due to the strong stabilization of the s electrons by the high nuclear charge and relativistic effects. The intermolecular distances Pb⋅⋅⋅S of 365(1) pm and Pb⋅⋅⋅O of 352(1) pm suggest secondary interactions that group the molecules into pairs. The major difference between the molecules of both complexes is the conformation at the N atom of the (thio)morpholine heterocycle: In **C1** the exocyclic atom C22 is in the axial position, while the corresponding atom in **C2** takes the equatorial position. This leads to small differences in packing, but the sequences along *a*=822 pm in **C1** and *b*=801 pm in **C2** are very similar (Figure 4).

### Bulk characterization of the ligand and the lead(II) complex

**Figure 5:**
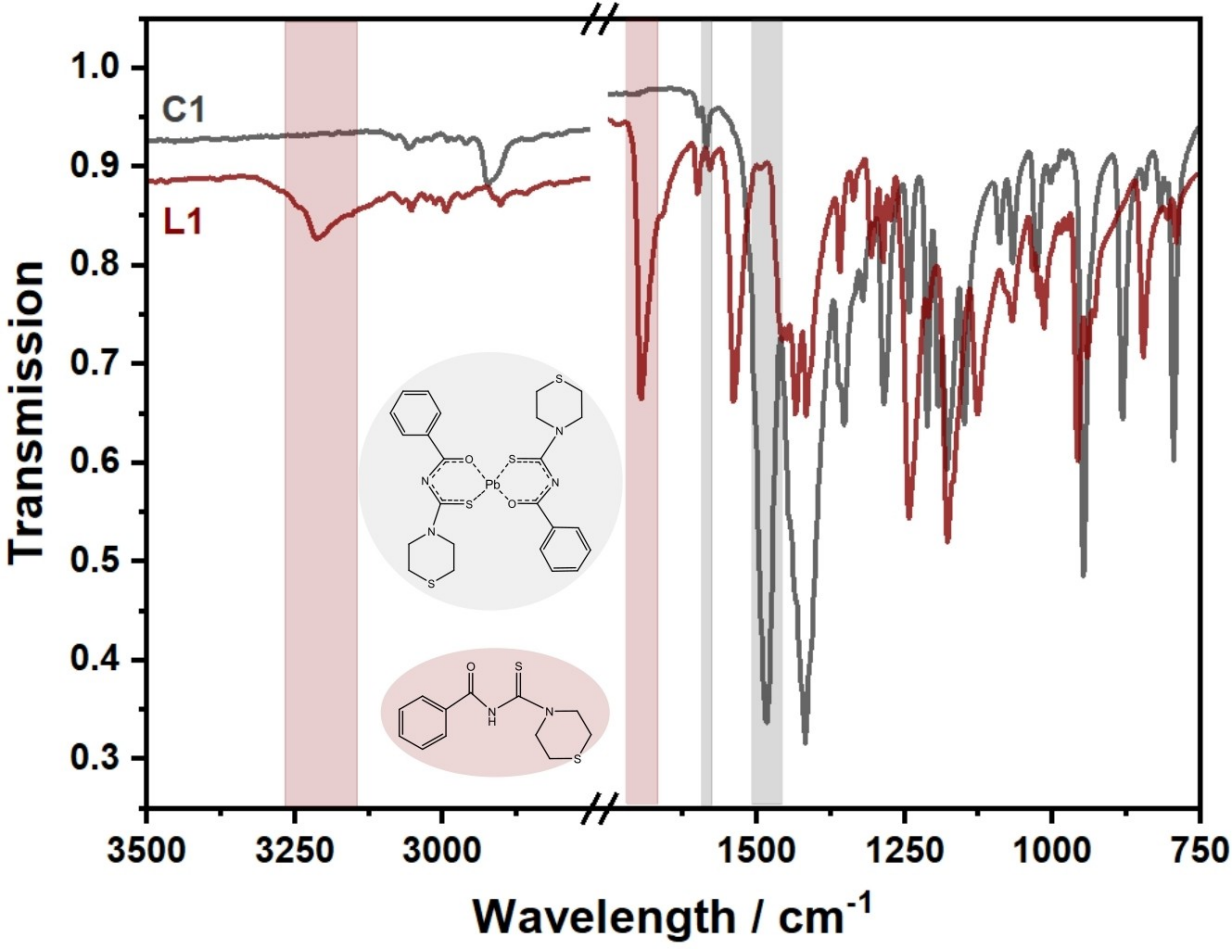
FTIR spectra of **C1** and **L1**.

The yellow and brown powders of **L1** and **C1** were produced in good yields, and the expected formulae were obtained as confirmed by microanalysis. The IR stretching of the N−H group close to the carbonyl group is related to the medium‐intensity peak around 3212 cm^−1^ for the ligand (Figure 5). When the ligand is bonded to the lead(II) atom by the action of a base, the N−H bond is replaced by the C=N bond. The C=N bond is responsible for the appearance of a new peak in the IR spectrum of **C1** at 1585 cm^−1^. The vibration of the C=O unit of the carbonyl groups, which shifted to lower wavenumbers upon complexation, is responsible for the strong band of the ligand which appears at 1695 cm^−1^. The deprotonation process resulted in the shift of the C=O stretching vibration from 1695 to 1483 cm^−1^, which is consistent with literature values,[^1^, ^42^] demonstrating the coordination to the central lead atom via the oxygen atom of the carbonyl group.

**Figure 6:**
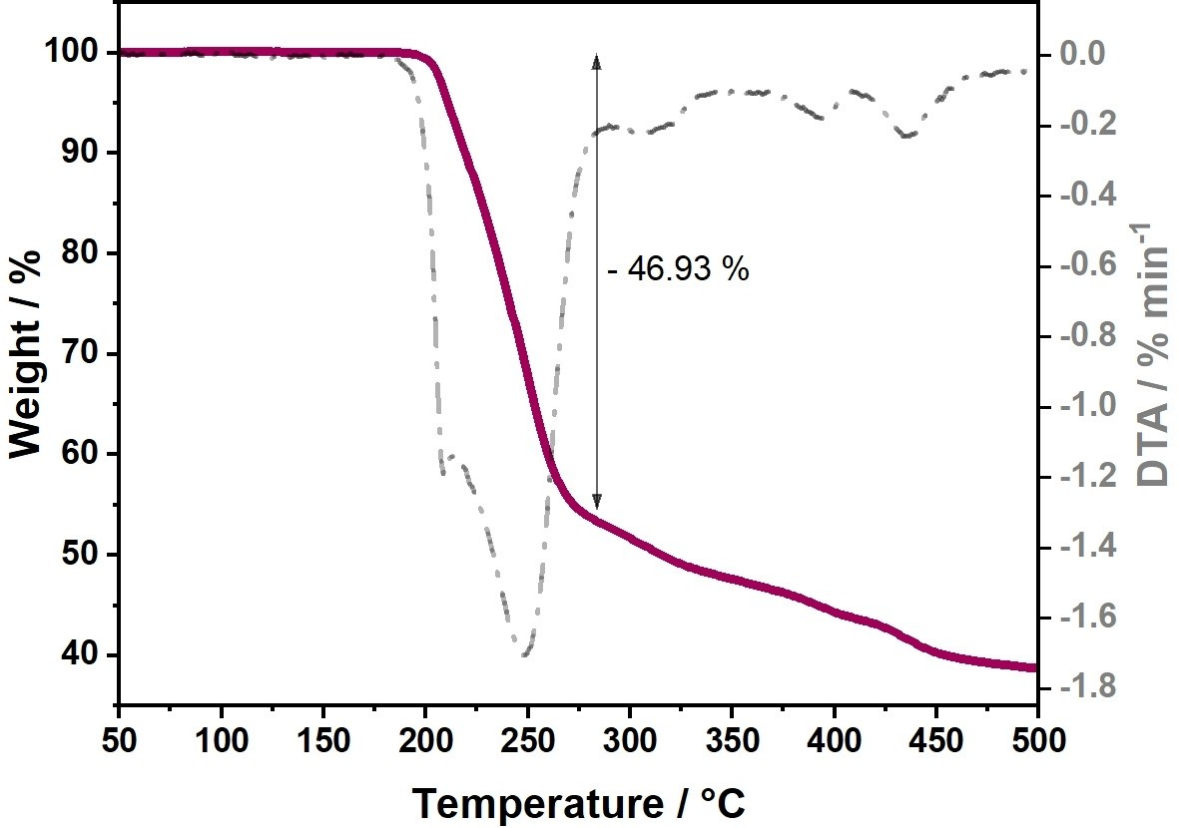
Thermogravimetry of **C1**, showing its thermal decomposition behavior in synthetic air.

TG‐DTA of **C1** was performed under synthetic air to understand the decomposition behavior of the complex to establish a protocol for thin film fabrication. TG‐MS was performed to gain an understanding of the decomposition mechanism and fragmentation products of the complex during the processing. The TG reveals an almost 47 % weight loss up to 280 °C, later trailing into smaller steps, that is, a gradual decomposition process (Figure 6). The mass spectrum reveals that the heaviest organic fragments, including urea (*m*=60 g mol^−1^) among others are lost during this first step. CO_2_ (*m*=44 g mol^−1^) seems to be evolving at various stages throughout the decomposition process and sulfur is removed mainly as SO_2_ (*m*=64 g mol^−1^) around 300 °C (Figure S1). We also performed thermal analysis of the complex under inert conditions (stream of argon) to check the extent of influence of the atmosphere on the decomposition process (Figure S2). We note that while the nature of the TG curve looks similar for the first main step, it appears to differ in the later stages of the decomposition process.

This is to be expected, as the mass spectrum indicates that the primary decomposition products at higher temperatures under synthetic air are gaseous oxides, which cannot be formed to this extent under inert conditions. Most importantly, we note that there is a downshift in temperature by about 80 °C of completion of the first decomposition step, which goes to show the significant impact of the environment on the decomposition temperature of the complex. This, combined with the fact that the presence of water in the processing atmosphere is still not accounted for, implies that the fabrication of thin films can be attempted at quite a low temperature.

### Spin‐coated PbS thin films

**Figure 7:**
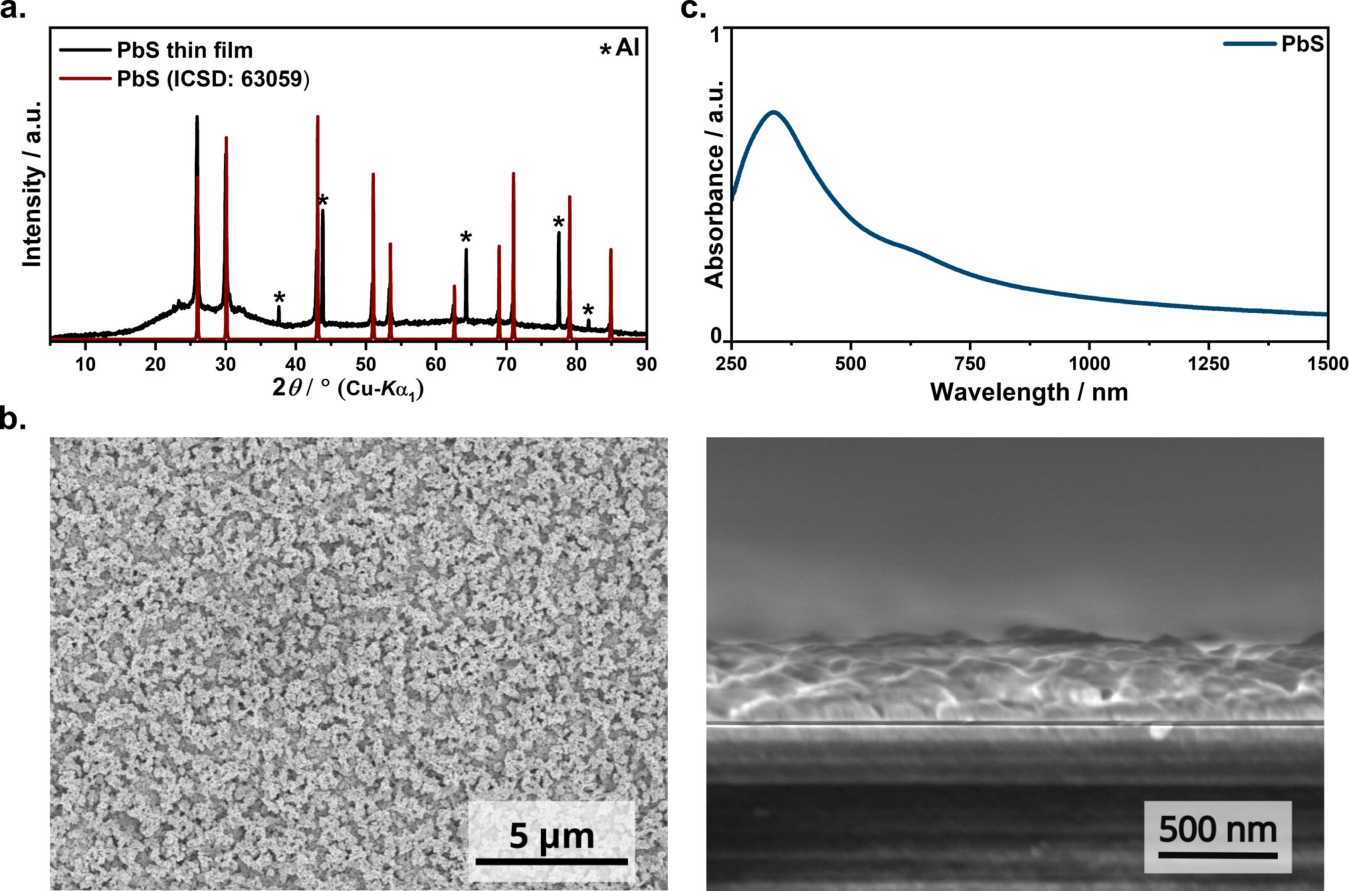
(a) PXRD of spin‐coated PbS thin films. The diffuse scattering is caused by the glass substrate. (b) Top view and cross‐section SEM images of spin‐coated thin films annealed at 250 °C for 30 min. (c) UV–Vis‐NIR absorption spectrum of a spin‐coated PbS film.

In previous work, aerosol‐assisted chemical vapour deposition (AACVD) was used to deposit PbS thin films from complex **C2**. The films were synthesized by adjusting experimental parameters such as the substrate temperature (from 350 to 450 °C).^[1]^ AACVD incorporates solution‐based precursor delivery employing solvents from which an aerosol can be produced. The aerosol is subsequently carried into the reaction chamber, where a heterogeneous or homogeneous breakdown mechanism under reaction temperature results in the development of films. However, this method requires expensive, specialized equipment. Spin‐coating was used to demonstrate the suitability of **C1** as a molecular precursor to obtain nanostructured PbS thin films in ambient atmosphere. PbS has a propensity towards oxidation in air, which is commonly employed as roasting in the extraction of lead from its ore, galena. This compelled us to carefully optimize the annealing temperature and time in order to ensure complete decomposition of the precursor whilst preventing further oxidation of the so‐formed PbS. Based on the argument for the decomposition temperature presented above, we tested the temperatures 200 °C, 250 °C and 300 °C for the annealing step. The optimal temperature was found to be 250 °C. It was observed that at temperatures higher than this, other oxidized by‐products such as PbO and Pb_3_O_2_Cl_2_ (chloroform as chlorine source) are formed in addition to PbS. Although TGA indicates that the decomposition of the complex is not complete at this temperature, the diffractogram of the thin film fabricated by annealing at 250 °C in air for 30 min shows reflections of PbS only (Figure 7a). Such complete decomposition of complex at temperatures lower than expected is frequently seen when fabricating thin films compared to bulk products due to the high surface energy of the substrates and less quantity of material.^[43]^

The diffractogram of the so‐formed PbS thin films is given in Figure 7a and the most dominant reflections are marked and assigned to the cubic phase of PbS (ICSD: 63059). Moreover, no other reflections of **L1**, **C1** or other decomposition products are observed, suggesting monophasic PbS film formation.^[44]^ The high crystallinity of PbS in the film is evidenced by the sharp and high intensity reflections.

The morphology of the PbS thin films was studied using SEM analysis (Figure 7b). The top‐view image shows long‐range uniformity and reasonably dense nature of the film, and the cross‐sectional view demonstrates the adhesion of the film to the substrate. PbS forms cuboidal particles of fairly homogenous size distribution between 80 and 150 nm which appear to form agglomerates. In order to confirm the composition of the spin‐coated films, EDX analysis was carried out. A representative EDX spectrum shows peaks from lead and sulfur along with other elements that originate from the glass substrate (Figure S3). The average of measurements over multiple areas of the film revealed a Pb : S ratio of 1.23, implying a slightly reduced sulfur content. This ratio is in accordance to the literature values with the sulfur deficiency being commonly noted in ambiently processed thin films of PbS.

Furthermore, there is an inherent error in the exact estimation of S‐content in thin films prepared on glass substrates due to the presence of high amounts of other elements such as Si, O, Na, Mg, Ca, etc. from the glass substrate. Although PXRD and EDX results prove the phase purity of the PbS thin film, we performed FTIR analysis on thin films of C1 and PbS (Figure S4). The spectra further proved that there are no residues of C1 or any other organic moieties in the PbS thin film within the detection limit of the instrument.

The optical absorption of the as‐deposited thin films was studied using UV–Vis‐NIR spectroscopy in transmission mode (Figure 7c). The absorption is typical of PbS and in agreement with the black color of the sample. The sample absorbs over a broad range in the visible and UV regions with an absorption maximum in the blue region. Furthermore, the spectrum is strongly blue‐shifted compared to bulk PbS which can be attributed to the smaller particle size in the thin films. The same trend was observed in previous reports by Savin et al.^[45]^ and Vankhade et al.^[46]^ Since the morphology has an influence on the optical properties, these results are different from those obtained in the previous work because PbS thin films deposited using the complex **C2** had given a mixture of spherical and cubic along some rod‐shaped particles with absorptions in the near visible range (800‐900 nm). Hence, the morphology and the optical properties have been considerably improved.

## Conclusion

A *N*‐(thiomorpholine‐4‐carbothioyl)benzamide ligand and its lead(II) complex have been synthesized, characterized and their crystal structures determined using single‐crystal diffraction methods. The lead(II) complex was successfully used as single‐source molecular precursor to deposit PbS thin films in ambient atmosphere by spin‐coating at temperatures much lower than the bulk decomposition temperature. Analyses confirmed the formation of crystalline PbS in the cubic phase. As expected, the spin‐coated PbS films showed a blue shift in their absorption maximum as compared to bulk PbS. The deposited thin films can potentially be used in photocatalysis and opens the way for further investigation on the photocatalytic properties of these PbS thin films. Moreover, the molecular precursor approach can be further extended by complexing this ligand with other metal ions for synthesis of corresponding metal sulfides, a vital class of semiconductors.

## Experimental Section

**Reagents**. Benzoyl chloride 99 %, thiomorpholine 99 %, potassium thiocyanate 98.5 %, lead acetate trihydrate 99.5 % were obtained from Sigma–Aldrich, hydrochloric acid 37 %, ethanol (EtOH) 98 % and acetone 99.5 % from Merck and dichloromethane (DCM) 99.8 % and chloroform 99.8 % from Fischer scientific. All the reagents were used as purchased without any further purification.

**Synthesis**. For the synthesis of **L1**, benzoyl chloride (2 mL; 17.22 mmol) was dissolved in 50 mL of acetone and added dropwise to a suspension of potassium thiocyanate (1.67 g; 17.22 mmol) in 30 mL of acetone. After 30 min of heating to reflux, the reaction mixture was cooled to room temperature. A solution of thiomorpholine (1.74 mL; 17.22 mmol) in 10 mL of acetone was added and the resulting mixture stirred for 2 h. The solid product formed was filtered off, washed with demineralized water, and purified by recrystallization in a 1 : 1 v/v EtOH : DCM mixture after adding 300 mL of 0.1 m HCl to the resulting solution. **C1** was synthesized by dissolving lead(II) acetate trihydrate (0.76 g; 2.00 mmol) in 10 mL of water and adding it dropwise to a solution of **L1** (1.00 g; 4.00 mmol) in 50 mL of EtOH at room temperature. The resulting mixture was stirred for 1 h and the solid product obtained was filtered off and recrystallized in 1 : 1 v/v EtOH : DCM mixture.

*N*‐(thiomorpholine‐4‐carbothioyl)benzamide (**L1**): Yellow. Yield: 80 %, m. p. 145 °C. Elem. anal. calcd. for C_12_H_14_N_2_OS_2_ (%): C, 54.11; H, 5.30; N, 10.52; S, 24.07. Found: C, 53.68; H, 5.16; N, 10.37; S, 26.65. IR (cm^−1^): ν(N−H) 3212 (br), ν(C=O) 1695 (s), ν(C=S) 1014 (s). ^1^H NMR (600 MHz, aceton‐d_6_): δ=8.0 (s, 1H, NH), 7.64 (m, 2H, C_6_H_5_), 7.53 (d, 1H, C_6_H_5_), 7.51 (m, 2H, C_6_H_5_), 3.90 (s, 4H, CH_2_), 2.90 (s, 4H, CH_2_).

Bis(N‐(thiomorpholine‐4‐carbothioyl)benzamidate)lead(II) (**C1**): Brown. Yield: 90 %, m. p. 232 °C. Elem. anal. calcd. for C_24_H_26_N_4_O_2_PbS_4_ (%): C, 39.06; H, 3.55; N, 7.59; S, 17.38. Found: C, 38.20; H, 3.42; N, 7.07; S, 17.24. IR (cm^−1^): ν(C=N) 1585 (s), ν(C=O) 1483 (s). ^1^H NMR (600 MHz, CDCl_3_ ): δ=8.20 (m, 2H; 2‐C_6_H_5_), 7.53 (d, 1H, 2‐C_6_H_5_), 7.45 (m, 2H, 2‐C_6_H_5_), 4.50 (m, 4H, 2‐CH_2_), 3.90 (m, 4H, 2‐CH_2_).

**Deposition of PbS thin films**. PbS thin films were prepared by thermal decomposition of a 0.2 mol L^−1^ solution of complex **C1** in chloroform. Glass substrates were cut into 1.5×1.5 cm^2^ followed by sequential ultrasonication in demineralized water, acetone and isopropanol for 10 min each to clean them thoroughly. They were dried in an oven at 60 °C. The spin‐coating process was carried out using 40 μL of the precursor solution with a spin program for 20 s at 2000 rpm and maximum acceleration (Polos SPS150i spin coater). The resulting film was then dried at 100 °C on a hotplate for 30 s to evaporate the solvent. This process was repeated for a total of 4 coating cycles. The substrates were then transferred to a muffle furnace, held at 250 °C, for the final annealing step for 30 min. All fabrication steps, storage and handling of PbS thin films were done in ambient atmosphere.

**Chemical analysis**. Elemental microanalysis was performed with a Flash Smart Elemental Analyzer 2021.FLS 0186 CHNS/O+MVC.

**Spectroscopy**. Infrared spectra of the compounds were measured in range the 4000 to 400 cm^−1^ using a KBr disc on a single reflectance ATR spectrometer.

^1^H NMR data were recorded at ambient temperature (∼23 °C) on a Bruker AV‐III 600 spectrometer operating at 600 MHz. Chemical shifts *δ* are given in ppm relative to TMS, coupling constants *J* are given in hertz. The solvent signals were used as reference (^1^H: *δ*_H_ 7.260 ppm residual CHCl_3_ or 2.050 ppm acetone‐d_5_).

UV–Vis‐NIR spectra of PbS thin films were acquired in the transmission mode in the range of 250 to 2000 nm using a Cary 5000 UV–Vis‐NIR spectrometer. Spin‐coated samples prepared on high quality 1×1 inch^2^ quartz substrates (Techinstro Ltd.) were used for the measurements.

**Thermal analysis**. TG‐DTA was carried out using a DSC (Jupiter) TGA device up to 500 °C at a heating rate of 10 °C min^−1^ employing an empty aluminum crucible as reference under synthetic air (80 % N_2_+20 % O_2_) and upto 700 °C at a heating rate of 30 °C min^−1^ under argon gas flow. TG‐MS was carried out up to 500 °C at a heating rate of 10 °C min^−1^ under synthetic air (80 % N_2_+20 % O_2_) on the same device.

**Electron microscopy**. Scanning electron microscopy (SEM) was performed using an SU8020 electron microscope (Hitachi) with a triple detector system for secondary and low‐energy backscattered electrons. An acceleration voltage of *U*_a_=2 kV with a current of 5 μA was used to acquire top‐view images. Samples of PbS thin film prepared on glass substrates were measured under N_2_‐cooling to reduce charging effects.

The composition of the film was determined by semi‐quantitative energy dispersive X‐ray (EDX) analysis (*U*_a=_15 kV) using a Silicon Drift Detector (SDD) X‐MaxN (Oxford Instruments) and the data was processed with the AZtec software package (Oxford Instruments, 2013). Samples were sputtered with approximately 5 nm gold layer before EDX measurements.

**X‐ray diffraction**. Powder X‐ray diffraction (PXRD) of the PbS thin films was performed in Bragg–Brentano geometry on a PANalytical Empyrean diffractometer equipped with a Johansson monochromator using Cu−*K*α_1_ radiation (*λ*=154.056 pm).

Suitable crystals for single‐crystal X‐ray diffraction (SCXRD) were obtained by slow evaporation of small amounts of the compounds dissolved in a mixture of ethanol and dichloromethane (1 : 1). The obtained crystals were apparently colorless despite of the color of the powder. The rectangular prismatic crystals were too large for SCXRD and hence were cut to a proper size with a scapel under perfluoropolyether oil. The fragments were mounted on a glass fiber and measured at 100(2) K using graphite‐monochromated Mo‐*K*α radiation (**λ**=71.073 pm) on a four‐circle Kappa Apex‐II diffractometer (Bruker), equipped with a CCD detector. Data reduction and a semi‐empirical (multi‐scan)^[47]^ absorption correction were carried out using the APEX3 suite.[^48^, ^49^] An initial molecular model was obtained with SHELXT^[50]^ and subsequently refined against *F*_o_^2^ with SHELXL.^[51]^ Anisotropic displacement parameters were refined for all non‐hydrogen atoms. Hydrogen positions were localized in the residual electron density map and refined using a riding model, with exception of H1 of **L1**, which was refined without constraints. Diamond^[52]^ was used for the creation of the graphics of the crystal structure and OLEX2^[53]^ for visualization during the refinement.

Deposition Numbers 2169985 (for **L1**), 2170099 (for **C1**) contain the supplementary crystallographic data for this paper. These data are provided free of charge by the joint Cambridge Crystallographic Data Centre and Fachinformationszentrum Karlsruhe Access Structures service.

## Funding

This research received financial assistance from the African‐German Network of Excellence in Science (2021 AGNES Grant for Juniors Researchers) in collaboration with the Alexander von Humboldt Foundation (AvH) and the German Ministry of Education and Research (BMBF).

## Conflict of interest

The authors declare no conflict of interest.

1

## Supporting information

As a service to our authors and readers, this journal provides supporting information supplied by the authors. Such materials are peer reviewed and may be re‐organized for online delivery, but are not copy‐edited or typeset. Technical support issues arising from supporting information (other than missing files) should be addressed to the authors.

Supporting Information

## Data Availability

The data that support the findings of this study are available in the supplementary material of this article.
