# Supplementary material for: Thiourea‐Derived Single‐Source Molecular Precursor For Spin‐Coated PbS Thin Films
Source: ChemistryOpen. 2023 Apr 14;12(4):e202300045. doi: 10.1002/open.202300045 (PMC10104949; doi:10.1002/open.202300045)
Supplement: Supplementary file 1 — Supporting Information [file OPEN-12-e202300045-s001.pdf]

ChemistryOpen

Supporting Information

Thiourea-Derived Single-Source Molecular Precursor For Spin-Coated PbS Thin Films

Kevin I. Y. Ketchemen,* Vaidehi Lapalikar, Eduardo Carrillo-Aravena, Linda D. Nyamen, Peter T. Ndifon, and Michael Ruck

Contents

I. Crystallographic data 2
1. Crystal data and structure refinement for **L1** and **C1**. 2
2. SHELX result file obtained from the refinement of **L2** 3
3. Crystallographic data for **L1** 5
4. Crystallographic data for **C1** 8
II. Thermal analysis of **C1** 13
III. EDX spectrum of PbS thin film 14
IV. FT-IR spectra of PbS thin film 14

I. Crystallographic data

1. Crystal data and structure refinement for **L1** and **C1**.

Table S1. Crystal data and structure refinement for **L1** and **C1**.

Chemical Formula	$C_{12}H_{14}N_2OS_2$ (**L1**)	$C_{24}H_{26}N_4O_2S_4Pb$ (**C1**)
Molar mass	266.37 g mol^{-1}	737.92 g mol^{-1}
Crystal system	Triclinic	Triclinic
Space group, Z	$P\bar{1}$ (no. 2), 2	$P\bar{1}$ (no. 2), 2
Temperature	100(2) K	100(2) K
Lattice parameters	*a* = 558.69(2) pm<br>*b* = 1060.77(3) pm<br>*c* = 1067.94(3) pm<br>α = 100.135(2)°<br>β = 94.915(2)°<br>γ = 95.358(2)°	*a* = 821.84(2) pm<br>*b* = 1202.03(3) pm<br>*c* = 1449.89(4) pm<br>α = 73.332(1)°<br>β = 83.056(1)°<br>γ = 71.651(1)°
Volume	616.93(3) · 10^6 pm^3	1301.63(6) · 10^6 pm^3
Calculated density	1.434 g cm^{-3}	1.883 g cm^{-3}
Diffractometer	Bruker Kappa APEX II CCD, graphite monochromator	
Radiation type	λ(Mo-K_α) = 71.073 pm	
Data range	1.95° ≤ θ ≤ 36.38°<br>–9 ≤ *h* ≤ 9<br>–17 ≤ *k* ≤ 17<br>–17 ≤ *l* ≤ 17	1.85° ≤ θ ≤ 36.41°<br>–13 ≤ *h* ≤ 13<br>–20 ≤ *k* ≤ 20<br>–24 ≤ *l* ≤ 24
Crystal size	0.120 × 0.091 × 0.059 mm^3	0.129 × 0.049 × 0.031 mm^3
Absorption correction	Multi-scan	
Absorption coefficient	0.42 mm^{-1}	6.83 mm^{-1}
T_{min}, T_{max}	0.71, 0.75	0.46, 0.75
Meas. reflections	32731	92339
Unique reflections	6001	12676
Reflections $I > 2\sigma(I)$	5197	11804
R_{int}	0.026	0.031
R_σ	0.019	0.020
Data / parameters	6001 / 158	12676 / 316
$R_1(F_o > 4\sigma(F_o))$	0.027	0.016
R_1(all)	0.034	0.019
wR_2(all)	0.068	0.030
Goof	0.92	1.09
$\Delta\rho_{max}$, $\Delta\rho_{min}$	0.54, –0.28 *e* 10^{-6} pm^{-3}	0.89, –0.56 *e* 10^{-6} pm^{-3}

2. SHELX result file obtained from the refinement of **L2**

```
TITL namiko229
REM N-(morpholine-4-carbothioyl)benzamide in P21/n at 296 K
REM Refined from data found in CCDC
REM Reference N. Ozdemir CCDC 2100916



CELL 0.71073 5.0731 11.912 20.9581 90 95.208 90
ZERR 4 0.0004 0.0012 0.0017 0 0.007 0
LATT 1
SYMM 0.5-X,0.5+Y,0.5-Z
SFAC C H N O S
UNIT 48 56 8 8 4
TEMP 23
SIZE 0.38 0.4 0.79
L.S. 10
BOND $H
FMAP -2
DFIX 0.88 N1 H1
ACTA
WGHT    0.070000    0.500000
FVAR       0.77903
S1    5    0.299201    0.322876    0.256540    11.00000    0.11044    0.07444 =
         0.07866    0.00856    0.00817   -0.01609
C1    1    0.458223    0.445246    0.258378    11.00000    0.04709    0.07041 =
         0.05246    0.00154    0.00305    0.00725
O1    4    0.880703    0.514902    0.342377    11.00000    0.04624    0.24121 =
         0.08611   -0.07406    0.00560    0.01925
N1    3    0.441579    0.516733    0.312246    11.00000    0.04423    0.07822 =
         0.05067   -0.00218    0.01078    0.00516
H1    2    0.287749    0.517435    0.327514    11.00000    0.08834
O2    4    0.695760    0.583777    0.094819    11.00000    0.22073    0.13198 =
         0.07332   -0.02475    0.07235   -0.07635
C2    1    0.658619    0.543606    0.353225    11.00000    0.04844    0.10029 =
         0.05677   -0.01361    0.01191    0.00639
N2    3    0.596113    0.485043    0.212757    11.00000    0.06434    0.08013 =
         0.05367   -0.01372    0.01650   -0.00884
C3    1    0.606344    0.608965    0.411177    11.00000    0.06597    0.07594 =
         0.04931   -0.00246    0.01881    0.00275
C4    1    0.415109    0.685089    0.412935    11.00000    0.14787    0.07772 =
         0.08588   -0.01360   -0.01079    0.04871
AFIX  43
H4    2    0.298951    0.697868    0.376734    11.00000   -1.20000
AFIX   0
C5    1    0.388484    0.745476    0.468639    11.00000    0.19955    0.11163 =
         0.12568   -0.04835   -0.01313    0.08057
AFIX  43
H5    2    0.252138    0.797592    0.469012    11.00000   -1.20000
AFIX   0
C6    1    0.548439    0.731677    0.520654    11.00000    0.13525    0.09035 =
         0.08681   -0.02979    0.05029   -0.00814
AFIX  43
H6    2    0.530865    0.775618    0.556701    11.00000   -1.20000
AFIX   0
C7    1    0.734755    0.654535    0.521128    11.00000    0.22306    0.21348 =
         0.06414   -0.04956   -0.01286    0.08321
AFIX  43
H7    2    0.841878    0.639786    0.558582    11.00000   -1.20000
AFIX   0
C8    1    0.770594    0.595258    0.465409    11.00000    0.18560    0.26628 =
         0.08562   -0.07859   -0.04038    0.12983
AFIX  43
H8    2    0.910719    0.544936    0.465317    11.00000   -1.20000
AFIX   0
C9    1    0.707209    0.599039    0.210491    11.00000    0.09793    0.10373 =
         0.07162   -0.02438    0.03472   -0.04049
AFIX  23
H9A   2    0.898904    0.595883    0.216574    11.00000   -1.20000
```

```
H9B   2    0.644859    0.644686    0.244378    11.00000   -1.20000
AFIX   0
C10   1    0.620671    0.649439    0.146523    11.00000    0.21823    0.10125 =
         0.06336   -0.01081    0.05317   -0.05083
AFIX  23
H10A  2    0.429659    0.657669    0.142566    11.00000   -1.20000
H10B  2    0.697415    0.723728    0.144084    11.00000   -1.20000
AFIX   0
C11   1    0.573911    0.476560    0.096053    11.00000    0.13101    0.11162 =
         0.05948   -0.01837    0.03052   -0.03503
AFIX  23
H11A  2    0.621916    0.432321    0.060014    11.00000   -1.20000
H11B  2    0.383040    0.485362    0.092032    11.00000   -1.20000
AFIX   0
C12   1    0.659677    0.416003    0.157727    11.00000    0.08022    0.10538 =
         0.06703   -0.02740    0.02170   -0.00397
AFIX  23
H12A  2    0.569330    0.344333    0.158650    11.00000   -1.20000
H12B  2    0.848652    0.401870    0.160330    11.00000   -1.20000
AFIX   0
HKLF 4

REM  namiko229
REM R1 =  0.0670 for    1736 Fo > 4sig(Fo)  and  0.1067 for all    2966 data
REM    158 parameters refined using      1 restraints

END

WGHT      0.0750      0.4859

REM Highest difference peak  0.349,  deepest hole -0.318,  1-sigma level  0.047
Q1    1   0.7864  0.6471  0.4481  11.00000  0.05    0.35
Q2    1   0.6594  0.5610  0.4700  11.00000  0.05    0.35
Q3    1   0.8721  0.4226  0.3590  11.00000  0.05   -0.32
Q4    1   0.7041  0.6187  0.4653  11.00000  0.05   -0.30
Q5    1   0.1438  0.3080  0.2491  11.00000  0.05   -0.29
Q6    1   0.8476  0.5768  0.3292  11.00000  0.05    0.28
Q7    1   0.8659  0.4808  0.3486  11.00000  0.05    0.25
Q8    1   0.6341  0.5133  0.4878  11.00000  0.05   -0.23
Q9    1   0.7449  0.7158  0.4994  11.00000  0.05    0.22
Q10   1   0.9247  0.6772  0.4514  11.00000  0.05   -0.21
```

The .cif file with the embedded .hkl file used for refinement was taken from https://dx.doi.org/10.5517/ccdc.csd.cc28j5hh. Note that the atom numbering scheme was changed to match the one of **L1**

3. Crystallographic data for **L1**

Table S2. Coordinates and equivalent isotropic displacement parameters [/ pm^2] for non-hydrogen atoms in **L1** at 100(2) K. U_{eq} is defined as one-third of the trace of the orthogonalized U_{ij} tensor.

	x	y	z	U_{eq}
S(1)	0.35145(3)	0.67590(2)	0.02806(2)	118(1)
C(1)	0.1978(1)	0.60818(6)	0.13310(6)	89(1)
O(1)	0.3092(1)	0.51308(5)	0.36358(5)	136(1)
N(1)	0.2374(1)	0.48219(6)	0.14465(6)	99(1)
S(2)	-0.18645(4)	0.82524(2)	0.43434(2)	166(1)
C(2)	0.2993(2)	0.44275(6)	0.25957(6)	100(1)
N(2)	0.0358(1)	0.66456(6)	0.20138(6)	101(1)
C(3)	0.3612(2)	0.30779(7)	0.24335(6)	107(1)
C(4)	0.2480(2)	0.21194(7)	0.14389(7)	135(1)
C(5)	0.3188(2)	0.08842(7)	0.12989(8)	168(1)
C(6)	0.5005(2)	0.06066(8)	0.21464(8)	187(1)
C(7)	0.6105(2)	0.15595(8)	0.31489(8)	188(1)
C(8)	0.5399(2)	0.27936(7)	0.33006(7)	147(1)
C(9)	-0.1427(2)	0.59847(7)	0.26850(7)	113(1)
C(10)	-0.1284 (2)	0.65809(7)	0.40973(7)	139(1)
C(11)	0.0370(2)	0.87782(7)	0.33644(8)	160(1)
C(12)	0.0003(2)	0.79977(7)	0.20146(7)	131(1)

Table S3. Bond lengths [/ pm] and bond angles [/°] for **L1**.

S(1)-C(1)	168.7(1)	C(1)-N(2)-C(9)	125.40(6)
C(1)-N(2)	133.0(1)	C(12)-N(2)-C(9)	113.35(5)
C(1)-N(1)	139.9(1)	C(8)-C(3)-C(4)	120.32(6)
O(1)-C(2)	121.8(1)	C(8)-C(3)-C(2)	117.94(6)
N(1)-C(2)	139.1(1)	C(4)-C(3)-C(2)	121.74(6)
N(1)-H(1)	86(2)	C(5)-C(4)-C(3)	119.55(7)
S(2)-C(11)	180.6(1)	C(5)-C(4)-H(4)	120.2
S(2)-C(10)	181.0(1)	C(3)-C(4)-H(4)	120.2
C(2)-C(3)	148.8(1)	C(6)-C(5)-C(4)	120.17(7)
N(2)-C(12)	146.6(1)	C(6)-C(5)-H(5)	119.9
N(2)-C(9)	147.2(1)	C(4)-C(5)-H(5)	119.9
C(3)-C(8)	139.3(1)	C(5)-C(6)-C(7)	120.15(7)
C(3)-C(4)	139.5(1)	C(5)-C(6)-H(6)	119.9
C(4)-C(5)	139.1(1)	C(7)-C(6)-H(6)	119.9
C(4)-H(4)	95.0	C(8)-C(7)-C(6)	120.06(7)
C(5)-C(6)	138.9(1)	C(8)-C(7)-H(7)	120.0
C(5)-H(5)	95.0	C(6)-C(7)-H(7)	120.0
C(6)-C(7)	139.2(1)	C(7)-C(8)-C(3)	119.72(7)
C(6)-H(6)	95.0	C(7)-C(8)-H(8)	120.1
C(7)-C(8)	138.9(1)	C(3)-C(8)-H(8)	120.1
C(7)-H(7)	95.0	N(2)-C(9)-C(10)	112.37(6)
C(8)-H(8)	95.0	N(2)-C(9)-H(9A)	109.1
C(9)-C(10)	152.2(1)	C(10)-C(9)-H(9A)	109.1
C(9)-H(9A)	99.0	N(2)-C(9)-H(9B)	109.1
C(9)-H(9B)	99.0	C(10)-C(9)-H(9B)	109.1
C(10)-H(10A)	99.0	H(9A)-C(9)-H(9B)	107.9
C(10)-H(10B)	99.0	C(9)-C(10)-S(2)	112.15(5)
C(11)-C(12)	151.8(1)	C(9)-C(10)-H(10A)	109.2
C(11)-H(11A)	99.0	S(2)-C(10)-H(10A)	109.2
C(11)-H(11B)	99.0	C(9)-C(10)-H(10B)	109.2
C(12)-H(12A)	99.0	S(2)-C(10)-H(10B)	109.2
C(12)-H(12B)	99.0	H(10A)-C(10)-H(10B)	107.9
		C(12)-C(11)-S(2)	111.90(5)
N(2)-C(1)-N(1)	118.23(6)	C(12)-C(11)-H(11A)	109.2
N(2)-C(1)-S(1)	124.39(5)	S(2)-C(11)-H(11A)	109.2
N(1)-C(1)-S(1)	117.32(5)	C(12)-C(11)-H(11B)	109.2
C(2)-N(1)-C(1)	124.73(6)	S(2)-C(11)-H(11B)	109.2
C(2)-N(1)-H(1)	114.2(9)	H(11A)-C(11)-H(11B)	107.9
C(1)-N(1)-H(1)	112.3(9)	N(2)-C(12)-C(11)	110.98(6)
C(11)-S(2)-C(10)	96.37(3)	N(2)-C(12)-H(12A)	109.4
O(1)-C(2)-N(1)	123.29(6)	C(11)-C(12)-H(12A)	109.4
O(1)-C(2)-C(3)	123.01(6)	N(2)-C(12)-H(12B)	109.4
N(1)-C(2)-C(3)	113.66(6)	C(11)-C(12)-H(12B)	109.4
C(1)-N(2)-C(12)	120.79(6)	H(12A)-C(12)-H(12B)	108.0

Table S4. Anisotropic displacement parameters [/ pm^2] for non-hydrogen atoms in **L1** at 100(2) K. The anisotropic displacement factor exponent takes the form: $-2\pi^2[h^2 a^{*2}U_{11} + ... + 2hka^*b^*U_{12}]$

	U_{11}	U_{22}	U_{33}	U_{23}	U_{13}	U_{12}
S(1)	128(1)	125(1)	121(1)	52(1)	44(1)	33(1)
C(1)	87(2)	93(2)	85(2)	18(2)	0(2)	9(2)
O(1)	156(2)	147(2)	100(2)	5(2)	-1(2)	41(2)
N(1)	122(2)	91(2)	88(2)	21(2)	16(2)	26(2)
S(2)	178(1)	173(1)	145(1)	-11(1)	53(1)	49(1)
C(2)	91(2)	111(3)	102(2)	32(2)	8(2)	16(2)
N(2)	101(2)	92(2)	112(2)	20(2)	25(2)	15(2)
C(3)	114(2)	106(2)	109(2)	34(2)	22(2)	23(2)
C(4)	157(3)	112(3)	137(3)	35(2)	7(2)	5(2)
C(5)	216(3)	105(3)	182(3)	27(2)	23(3)	9(2)
C(6)	221(3)	128(3)	230(4)	50(3)	44(3)	65(3)
C(7)	196(3)	178(3)	208(3)	58(3)	0(3)	88(3)
C(8)	153(3)	151(3)	144(3)	36(2)	-2(2)	51(2)
C(9)	91(2)	129(3)	118(3)	22(2)	20(2)	3(2)
C(10)	139(3)	169(3)	113(3)	22(2)	28(2)	28(2)
C(11)	175(3)	127(3)	168(3)	-10(2)	37(2)	14(2)
C(12)	159(3)	99(3)	144(3)	27(2)	36(2)	39(2)

Table S5. Coordinates and isotropic displacement parameters [/ pm^2] for hydrogen atoms in **L1** at 100(2) K.

	x	*y*	*z*	U_{iso}
H(1)	0.303(2)	0.444(2)	0.081(2)	20(3)
H(4)	0.1233	0.2309	0.0861	160
H(5)	0.2427	0.0229	0.0622	200
H(6)	0.5497	-0.0236	0.2042	220
H(7)	0.7342	0.1366	0.3730	230
H(8)	0.6131	0.3441	0.3993	180
H(9A)	-0.1151	0.5066	0.2600	140
H(9B)	-0.3070	0.6022	0.2276	140
H(10A)	0.0344	0.6520	0.4513	170
H(10B)	-0.2480	0.6087	0.4509	170
H(11A)	0.0275	0.9699	0.3330	190
H(11B)	0.2002	0.8695	0.3760	190
H(12A)	-0.1652	0.8049	0.1629	160
H(12B)	0.1163	0.8365	0.1488	160

4. Crystallographic data for **C1**

Table S6. Coordinates and equivalent isotropic displacement parameters [/ pm[2]] for non-hydrogen atoms in **C1** at 100(2) K. U_{eq} is defined as one-third of the trace of the orthogonalized U_{ij} tensor.

	x	*y*	*z*	U_{eq}
Pb	-0.03295(2)	0.05335(2)	0.12428(2)	120(1)
S(1)	0.25793(3)	0.05236(2)	0.01975(2)	118(1)
O(1)	-0.1299(1)	0.20108(8)	-0.02955(6)	152(2)
N(1)	0.0981(2)	0.27918(9)	-0.08742(7)	124(2)
C(1)	0.2177(2)	0.2099(1)	-0.02359(7)	102(2)
S(2)	0.43058(4)	0.40651(3)	0.11956(2)	166(1)
N(2)	0.3224(2)	0.26374(8)	-0.00241(7)	115(2)
C(2)	-0.0492(2)	0.2603(1)	-0.09454(8)	110(2)
C(3)	-0.1293(2)	0.3213(1)	-0.19038(8)	117(2)
C(4)	-0.0623(2)	0.4036 (2)	-0.26033(9)	173(2)
C(5)	-0.1359(2)	0.4571(2)	-0.34971(9)	206(2)
C(6)	-0.2758(2)	0.4281(2)	-0.37008(9)	192(2)
C(7)	-0.3436(2)	0.3471(2)	-0.30078(9)	189(2)
C(8)	-0.2712(2)	0.2943(2)	-0.21094(8)	153(2)
C(9)	0.2962 (2)	0.3951(1)	-0.04126(8)	144(2)
C(10)	0.2564(2)	0.4596(1)	0.03838(9)	157(2)
C(11)	0.4483(2)	0.2471(1)	0.14821(8)	134(2)
C(12)	0.4700(2)	0.2005(1)	0.05918(8)	122(2)
S(21)	-0.06598(4)	0.26440(3)	0.16354(2)	143(1)
O(21)	0.1425(2)	-0.01548(8)	0.26457(6)	141(2)
N(21)	0.1401(2)	0.14836(9)	0.31761(7)	138(2)
C(21)	-0.0057(2)	0.2289(1)	0.28241(8)	125(2)
S(22)	-0.40546(4)	0.26170(3)	0.48504(2)	191(1)
N(22)	-0.1019(2)	0.29829(9)	0.33898(7)	140(2)
C(22)	0.2163(2)	0.0447(1)	0.29394(7)	115(2)
C(23)	0.4044(2)	-0.0071(1)	0.30961(7)	112(2)
C(24)	0.4860(2)	0.0281(2)	0.37061(8)	149(2)
C(25)	0.6615(2)	-0.0227(2)	0.38283(9)	182(2)
C(26)	0.7567(2)	-0.1071(2)	0.33390(9)	189(2)
C(27)	0.6765(2)	-0.1411(2)	0.27206(9)	183(2)
C(28)	0.5008(2)	-0.0916(2)	0.26060(8)	144(2)
C(29)	-0.0682(2)	0.2661(2)	0.44168(8)	155(2)
C(30)	-0.1774(2)	0.1882(2)	0.50101(8)	182(2)
C(31)	-0.4043(2)	0.3105(2)	0.35516(8)	161(2)
C(32)	-0.2741 (2)	0.3793(1)	0.31412(8)	150(2)

Table S7. Bond lengths [/ pm] and bond angles [/°] for **C1**

Pb-O(21)	242.8(1)	C(23)-C(24)	139.5(2)
Pb-O(1)	245.5(1)	C(24)-C(25)	139.0(2)
Pb-S(1)	267.0(1)	C(24)-H(24)	95.0
Pb-S(21)	268.2(1)	C(25)-C(26)	138.7(2)
S(1)-C(1)	175.0(1)	C(25)-H(25)	95.0
O(1)-C(2)	126.3(1)	C(26)-C(27)	138.8(2)
N(1)-C(2)	132.1(1)	C(26)-H(26)	95.0
N(1)-C(1)	133.3(1)	C(27)-C(28)	138.9(2)
C(1)-N(2)	133.6(1)	C(27)-H(27)	95.0
S(2)-C(10)	180.1(1)	C(28)-H(28)	95.0
S(2)-C(11)	180.3(1)	C(29)-C(30)	151.8(2)
N(2)-C(12)	146.2(1)	C(29)-H(29A)	99.0
N(2)-C(9)	147.0(1)	C(29)-H(29B)	99.0
C(2)-C(3)	149.5(2)	C(30)-H(30A)	99.0
C(3)-C(8)	139.0(2)	C(30)-H(30B)	99.0
C(3)-C(4)	139.5(2)	C(31)-C(32)	151.7(2)
C(4)-C(5)	138.8(2)	C(31)-H(31A)	99.0
C(4)-H(4)	95.0	C(31)-H(31B)	99.0
C(5)-C(6)	138.7(2)	C(32)-H(32A)	99.0
C(5)-H(5)	95.0	C(32)-H(32B)	99.0
C(6)-C(7)	138.4(2)		
C(6)-H(6)	95.0	O(21)-Pb-O(1)	149.96(3)
C(7)-C(8)	138.9(2)	O(21)-Pb-S(1)	86.26(2)
C(7)-H(7)	95.0	O(1)-Pb-S(1)	76.49(2)
C(8)-H(8)	95.0	O(21)-Pb-S(21)	78.77(2)
C(9)-C(10)	151.8(2)	O(1)-Pb-S(21)	78.18(2)
C(9)-H(9A)	99.0	S(1)-Pb-S(21)	94.18(1)
C(9)-H(9B)	99.0	C(1)-S(1)-Pb	97.66(4)
C(10)-H(10A)	99.0	C(2)-O(1)-Pb	130.31(7)
C(10)-H(10B)	99.0	C(2)-N(1)-C(1)	125.9(1)
C(11)-C(12)	151.9(2)	N(1)-C(1)-N(2)	116.5(1)
C(11)-H(11A)	99.0	N(1)-C(1)-S(1)	123.01(8)
C(11)-H(11B)	99.0	N(2)-C(1)-S(1)	119.94(8)
C(12)-H(12A)	99.0	C(10)-S(2)-C(11)	97.01(5)
C(12)-H(12B)	99.0	C(1)-N(2)-C(12)	124.44(9)
S(21)-C(21)	174.6(1)	C(1)-N(2)-C(9)	121.14(9)
O(21)-C(22)	126.0(1)	C(12)-N(2)-C(9)	114.42(8)
N(21)-C(22)	132.9(2)	O(1)-C(2)-N(1)	127.5(1)
N(21)-C(21)	132.9(1)	O(1)-C(2)-C(3)	117.86(9)
C(21)-N(22)	134.7(1)	N(1)-C(2)-C(3)	114.6(1)
S(22)-C(31)	180.4(1)	C(8)-C(3)-C(4)	119.3(1)
S(22)-C(30)	181.4(1)	C(8)-C(3)-C(2)	119.7(1)
N(22)-C(32)	146.2(2)	C(4)-C(3)-C(2)	121.0(1)
N(22)-C(29)	146.5(1)	C(5)-C(4)-C(3)	120.2(2)
C(22)-C(23)	149.3(2)	C(5)-C(4)-H(4)	119.9
C(23)-C(28)	139.1(2)	C(3)-C(4)-H(4)	119.9

C(6)-C(5)-C(4)	120.1(2)	O(21)-C(22)-N(21)	125.8(1)
C(6)-C(5)-H(5)	119.9	O(21)-C(22)-C(23)	118.1(1)
C(4)-C(5)-H(5)	119.9	N(21)-C(22)-C(23)	116.01(9)
C(7)-C(6)-C(5)	120.0(2)	C(28)-C(23)-C(24)	119.4(1)
C(7)-C(6)-H(6)	120.0	C(28)-C(23)-C(22)	118.65(9)
C(5)-C(6)-H(6)	120.0	C(24)-C(23)-C(22)	121.9(1)
C(6)-C(7)-C(8)	120.0(2)	C(25)-C(24)-C(23)	119.8(2)
C(6)-C(7)-H(7)	120.0	C(25)-C(24)-H(24)	120.1
C(8)-C(7)-H(7)	120.0	C(23)-C(24)-H(24)	120.1
C(7)-C(8)-C(3)	120.4(2)	C(26)-C(25)-C(24)	120.5(2)
C(7)-C(8)-H(8)	119.8	C(26)-C(25)-H(25)	119.8
C(3)-C(8)-H(8)	119.8	C(24)-C(25)-H(25)	119.8
N(2)-C(9)-C(10)	111.52(9)	C(25)-C(26)-C(27)	119.9(2)
N(2)-C(9)-H(9A)	109.3	C(25)-C(26)-H(26)	120.0
C(10)-C(9)-H(9A)	109.3	C(27)-C(26)-H(26)	120.0
N(2)-C(9)-H(9B)	109.3	C(26)-C(27)-C(28)	119.7(2)
C(10)-C(9)-H(9B)	109.3	C(26)-C(27)-H(27)	120.1
H(9A)-C(9)-H(9B)	108.0	C(28)-C(27)-H(27)	120.1
C(9)-C(10)-S(2)	111.81(8)	C(27)-C(28)-C(23)	120.7(2)
C(9)-C(10)-H(10A)	109.3	C(27)-C(28)-H(28)	119.7
S(2)-C(10)-H(10A)	109.3	C(23)-C(28)-H(28)	119.7
C(9)-C(10)-H(10B)	109.3	N(22)-C(29)-C(30)	110.93(9)
S(2)-C(10)-H(10B)	109.3	N(22)-C(29)-H(29A)	109.5
H(10A)-C(10)-H(10B)	107.9	C(30)-C(29)-H(29A)	109.5
C(12)-C(11)-S(2)	112.72(8)	N(22)-C(29)-H(29B)	109.5
C(12)-C(11)-H(11A)	109.0	C(30)-C(29)-H(29B)	109.5
S(2)-C(11)-H(11A)	109.0	H(29A)-C(29)-H(29B)	108.0
C(12)-C(11)-H(11B)	109.0	C(29)-C(30)-S(22)	112.85(9)
S(2)-C(11)-H(11B)	109.0	C(29)-C(30)-H(30A)	109.0
H(11A)-C(11)-H(11B)	107.8	S(22)-C(30)-H(30A)	109.0
N(2)-C(12)-C(11)	111.10(9)	C(29)-C(30)-H(30B)	109.0
N(2)-C(12)-H(12A)	109.4	S(22)-C(30)-H(30B)	109.0
C(11)-C(12)-H(12A)	109.4	H(30A)-C(30)-H(30B)	107.8
N(2)-C(12)-H(12B)	109.4	C(32)-C(31)-S(22)	111.99(8)
C(11)-C(12)-H(12B)	109.4	C(32)-C(31)-H(31A)	109.2
H(12A)-C(12)-H(12B)	108.0	S(22)-C(31)-H(31A)	109.2
C(21)-S(21)-Pb	106.88(4)	C(32)-C(31)-H(31B)	109.2
C(22)-O(21)-Pb	127.38(7)	S(22)-C(31)-H(31B)	109.2
C(22)-N(21)-C(21)	125.4(1)	H(31A)-C(31)-H(31B)	107.9
N(21)-C(21)-N(22)	117.1(1)	N(22)-C(32)-C(31)	109.26(9)
N(21)-C(21)-S(21)	124.45(9)	N(22)-C(32)-H(32A)	109.8
N(22)-C(21)-S(21)	117.85(8)	C(31)-C(32)-H(32A)	109.8
C(31)-S(22)-C(30)	98.83(6)	N(22)-C(32)-H(32B)	109.8
C(21)-N(22)-C(32)	122.54(9)	C(31)-C(32)-H(32B)	109.8
C(21)-N(22)-C(29)	120.4(1)	H(32A)-C(32)-H(32B)	108.3
C(32)-N(22)-C(29)	113.66(9)		

Table S8. Anisotropic displacement parameters [/ pm^2] for non-hydrogen atoms in the **C1** at 100(2) K. The anisotropic displacement factor exponent takes the form: $-2\pi^2[h^2 a^{*2}U_{11} + ... + 2hka^*b^*U_{12}]$

	U_{11}	U_{22}	U_{33}	U_{23}	U_{13}	U_{12}
Pb	115(1)	156(1)	98(1)	-16(1)	-11(1)	-66(1)
S(1)	111(1)	99(1)	146(1)	-40(1)	-3(1)	-29(1)
O(1)	117(3)	206(4)	122(3)	-5(3)	-9(3)	-64(3)
N(1)	119(4)	128(4)	126(4)	-19(3)	-28(3)	-43(3)
C(1)	101(4)	111(4)	98(4)	-33(3)	9(3)	-37(3)
S(2)	241(1)	122(1)	154(1)	-20(1)	-62(1)	-73(1)
N(2)	117(4)	99(4)	133(4)	-15(3)	-34(3)	-42(3)
C(2)	110(4)	110(4)	113(4)	-41(3)	-11(3)	-21(3)
C(3)	119(4)	114(4)	112(4)	-40(3)	-23(3)	-11(3)
C(4)	160(5)	190(5)	151(5)	1(4)	-34(4)	-59(4)
C(5)	226(6)	211(6)	143(5)	15(4)	-29(4)	-61(5)
C(6)	235(6)	174(5)	132(5)	-31(4)	-72(4)	3(4)
C(7)	217(5)	181(5)	182(5)	-46(4)	-99(4)	-43(4)
C(8)	169(5)	151(5)	148(5)	-29(4)	-56(4)	-47(4)
C(9)	183(5)	112(5)	144(5)	7(4)	-54(4)	-71(4)
C(10)	167(5)	104(5)	190(5)	-18(4)	-27(4)	-35(4)
C(11)	159(5)	116(5)	125(4)	-6(4)	-36(4)	-48(4)
C(12)	88(4)	116(4)	159(5)	-33(4)	-31(3)	-20(3)
S(21)	168(1)	140(1)	87(1)	-17(1)	-11(1)	-7(1)
O(21)	156(4)	133(4)	138(3)	-9(3)	-52(3)	-57(3)
N(21)	132(4)	142(4)	133(4)	-44(3)	-21(3)	-20(3)
C(21)	133(4)	125(5)	111(4)	-23(4)	8(3)	-43(4)
S(22)	216(1)	259(2)	118(1)	-32(1)	0(1)	-116(1)
N(22)	157(4)	143(4)	100(4)	-35(3)	-5(3)	-11(3)
C(22)	137(4)	121(4)	79(4)	0(3)	-15(3)	-46(4)
C(23)	123(4)	108(4)	99(4)	-3(3)	-21(3)	-41(3)
C(24)	168(5)	139(5)	148(5)	-30(4)	-44(4)	-48(4)
C(25)	171(5)	201(6)	187(5)	-18(4)	-59(4)	-85(4)
C(26)	127(5)	225(6)	185(5)	-4(4)	-30(4)	-46(4)
C(27)	153(5)	198(6)	171(5)	-46(4)	-4(4)	-19(4)
C(28)	152(5)	148(5)	130(4)	-35(4)	-24(4)	-34(4)
C(29)	188(5)	178(5)	110(4)	-54(4)	-18(4)	-50(4)
C(30)	255(6)	181(5)	117(5)	-11(4)	-50(4)	-84(5)
C(31)	185(5)	175(5)	119(4)	-45(4)	-27(4)	-36(4)
C(32)	167(5)	112(5)	126(4)	-16(4)	0(4)	6(4)

Table S9. Coordinates and isotropic displacement parameters [/ pm^2] for hydrogen atoms in **C1** at 100(2) K.

	x	*y*	*z*	U_{iso}
H(4)	0.0341	0.4232	-0.2468	210
H(5)	-0.0904	0.5136	-0.3970	250
H(6)	-0.3251	0.4640	-0.4316	230
H(7)	-0.4396	0.3274	-0.3147	230
H(8)	-0.3191	0.2396	-0.1632	180
H(9A)	0.2002	0.4296	-0.0861	170
H(9B)	0.4008	0.4090	-0.0781	170
H(10A)	0.1516	0.4458	0.0750	190
H(10B)	0.2335	0.5481	0.0094	190
H(11A)	0.5478	0.2002	0.1895	160
H(11B)	0.3440	0.2330	0.1853	160
H(12A)	0.5754	0.2128	0.0224	150
H(12B)	0.4831	0.1123	0.0790	150
H(24)	0.4216	0.0866	0.4037	180
H(25)	0.7167	0.0005	0.4250	220
H(26)	0.8767	-0.1416	0.3427	230
H(27)	0.7417	-0.1980	0.2377	220
H(28)	0.4458	-0.1156	0.2189	170
H(29A)	-0.0936	0.3412	0.4631	190
H(29B)	0.0546	0.2212	0.4521	190
H(30A)	-0.1529	0.1688	0.5700	220
H(30B)	-0.1453	0.1107	0.4826	220
H(31A)	-0.3772	0.2385	0.3295	190
H(31B)	-0.5199	0.3634	0.3340	190
H(32A)	-0.2824	0.4094	0.2432	180
H(32B)	-0.2981	0.4501	0.3409	180

II. Thermal analysis of **C1**

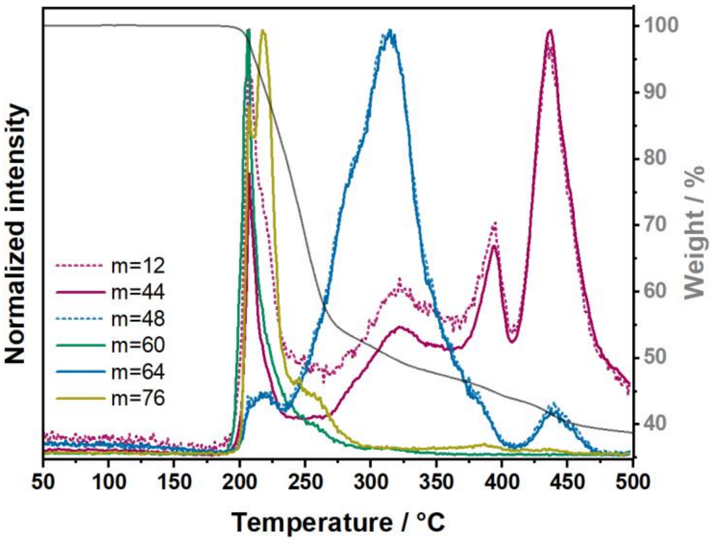

Figure S1. TG-MS of **C1** carried out under synthetic air showing plots of fragmentation products of different masses (given in the legend) overlaid on the TG curve for **C1**. Dotted lines represent signatures of fragmentation products identical to the corresponding plots in same-colored solid lines.

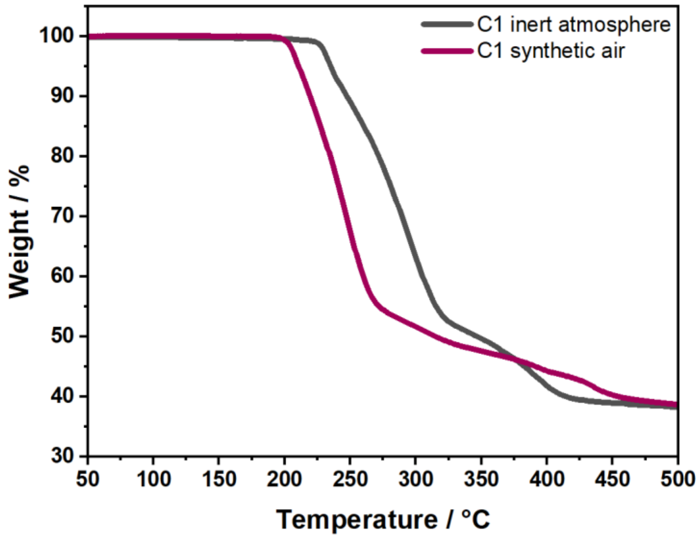

Figure S2. Comparison of TG of C1 carried out in inert atmosphere (stream of Ar gas) and synthetic air (80% N_2 + 20% O_2) showing a downshift by ~80 °C in the completion of the first step of the decomposition process.

III. EDX spectrum of PbS thin film

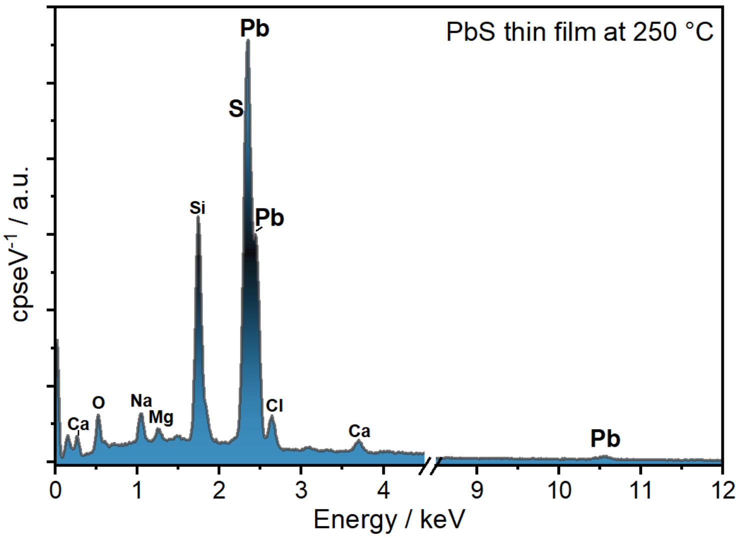

Figure S3. EDX spectrum of spin-coated PbS thin film annealed at 250 °C showing peaks of Pb and S along with peaks of other elements that originate from the glass substrate.

IV. FT-IR spectra of PbS thin film

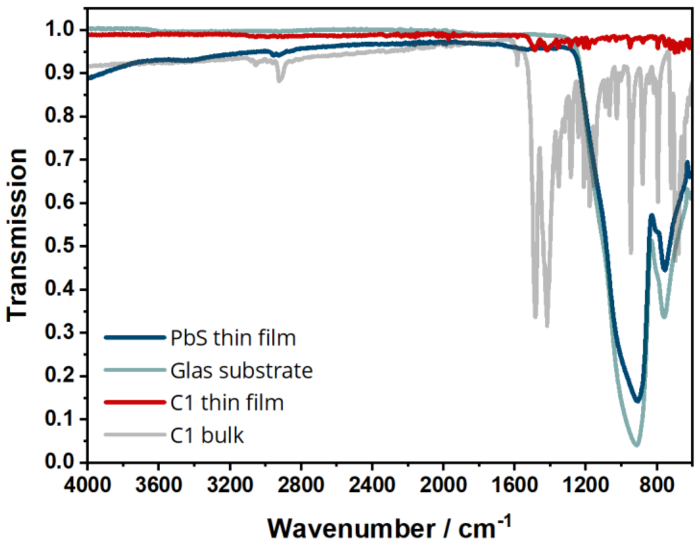

Figure S4. FT-IR spectrum of as-fabricated PbS thin film compared with thin film of C1 showing no peaks from C1 or any other organic residues. Spectra of the glass substrate and bulk C1 are included for reference.
